# Anthropogenic pollen indicators: Global food plants and Latin American human indicators in the pollen record

**DOI:** 10.1038/s41597-023-02613-1

**Published:** 2023-10-19

**Authors:** Suzette G. A. Flantua, Henry Hooghiemstra

**Affiliations:** 1 Department of Biological Sciences, University of Bergen and Bjerknes Centre for Climate Research, Bergen, Norway; 2 Institute for Biodiversity and Ecosystem Dynamics, University of Amsterdam, Science Park 904, 1098XH Amsterdam, The Netherlands

**Keywords:** Palaeoecology, History

## Abstract

Pollen-based evidence of human presence is crucial for reconstructing human history. However, information on the morphology of pollen grains of global food plants and regional pollen-based human indicators is scattered in the literature, leading to the risk of overlooking important evidence of human presence. To address this issue, we first compiled a comprehensive overview of 354 major food plants worldwide, creating a paleoecology-friendly format that includes their family, vernacular name, earliest known use, environmental preference, and geographical region. Moreover, we identified the sources of illustrations of their pollen grains for 209 out of 273 different genera of globally relevant food plants in 10 selected pollen atlases. Secondly, we compiled all human indicators from pollen-based paleoecological literature in Latin America (based on 750 references), providing an overview of 212 single-pollen type indicators and identified 95 crucial combinations of pollen types as “human indices”, and their corresponding references. Our review datasets aids in distilling human evidence from numerous fossil pollen records worldwide.

## Background & Summary

### The significance of identifying human evidence in fossil pollen records

Fossil pollen records have long been recognized as valuable sources of information for reconstructing past environments and the impact of human activities on these environments. Palynology, the study of pollen and spores of plants preserved in sediment records, provides critical evidence on the taxonomic composition of vegetation, and can be used to infer the presence and types of human-mediated changes, such as related to deforestation and agriculture^1–7^. Commonly used sources of information to trace human impact are fossil pollen (e.g.^8^), fossil fruits and seeds (e.g.^9^), phytoliths (e.g.^10^), and non-pollen palynomorphs (e.g.^11^), whereas records of charcoal fragments (e.g.^12^) have the potential to hint to human land use (e.g.^13^). The study of human evidence and impact derived from fossil pollen records is an interdisciplinary field that draws on expertise from botany, ecology, earth-sciences, archaeology and other related disciplines, and provides valuable insights into understanding the long-term history of human-environment relationships. With the increasing availability of fossil pollen records in open access databases such as the Neotoma Paleoecological Database^14^ and PANGAEA^15^, unique opportunities arise to synthesize human history over regional and continental scale.

### The diversity of anthropogenic pollen indicators

Anthropogenic pollen indicators are types of pollen grains found in sediment or fossil records that serve as evidence of human activities or human-induced changes in the environment^16,17^. These indicators are specific to certain plant species or groups that have been cultivated, introduced, or manipulated by human populations. The presence of these pollen types can provide insights into various aspects of human history and impact, such as the development and spread of agriculture, the expansion of human settlements, land use changes, and the introduction of non-native species. Behre^16,17^ offered the first overview lists of anthropogenic pollen indicators for north European records and numerous studies afterwards used and adapted its concept (e.g.^18–20^ and see references therein). Anthropogenic pollen indicators can be indicated by the presence and/or changes in abundance of adventives (i.e. plant species not native to a specific area), intentional or unintentional introductions (e.g. cultivated crops vs. weeds), apophytes (i.e. native plant species favoured by human activities, such as by vegetation disturbance^21^), and certain tree pollen^22^. Anthropogenic pollen indicators (hereafter ‘human indicators’) are an important tool in paleoecology and archaeological studies as they help reconstruct past human activities and their influence on vegetation.

### Challenges in identifying human indicators in fossil pollen records

Paleoecologists often familiarize themselves with the commonly recognized human indicators found in pollen diagrams, such as introduced plants, crop species, or those that were widespread in a particular region (e.g.^23,24^). Likewise, some paleoecologists are familiar with human indicators based on enthobotanical evidence relevant for the region (^9,25,26^). To support the identification of pollen types of interest, reference materials that describe the pollen morphology and provide illustrations by light microscope (LM) photographs and/or scanning electron microscope (SEM) images are of crucial relevance. However, during the analysis of fossil pollen datasets and diagrams, palynologists may not always be aware of which higher ranked taxonomic levels (plant families and genera) contain potential evidence of human presence, leading to the potential oversight of significant indicators and underestimation of the extent and nature of human activity. Unfortunately, the information on pollen-based human indicators in general and pollen morphology of food plants specifically is currently much dispersed throughout the literature. Lack of familiarity with human indicators poses the risk of missing crucial evidence of human presence.

### The crucial role of identifying food plants in fossil pollen records

The correct identification of food plants in particular is crucial for reconstructing human history. Firstly, it provides evidence of the subsistence strategies and diets of past human societies, and the interchange of crops and food-producing plants among societies (e.g.^26,27^). Secondly, it can provide insights into the relationship between human populations and their environments (e.g.^13,28^). For example, it can reveal the nature of deforestation related to the development of food production activities (e.g.^29,30^). Additionally, by examining changes in the use of food plants over space and time, we can trace the geographical distribution of food plants as a result of interchange among prehistorical societies. Moreover, it sheds light on the development and spread of agriculture, one of the most transformative innovations affecting the landscape in human history (e.g.^31,32^). By tracing the origins of food plants, we can gain a deeper understanding of the processes that led diversification of food production into modern day agriculture (e.g.^33,34^). Overall, the identification of food plants in fossil pollen records is a critical component of our understanding of human history.

### Objectives of this paper

This paper aims to facilitate the identification of human evidence in fossil pollen records worldwide through two approaches: a global perspective on food plants (Approach I.) and a continental focus on anthropogenic pollen indicators (i.e. human indicators) in Latin America (Approach II.). Our paper is structured covering first the global perspective of food related human indicators, followed by a continental perspective of all types of human indicators: **[I**.**a]** We first present a reference dataset that reviews the abundance of food plants by family and genus globally, based on Van Wyk’s book ‘Food plants of the World’^35^. **[I**.**b]** We guide the dataset user to the general pollen morphological literature where illustrations of pollen grains of these food plants can be found as LM photographs and/or SEM images. **[I**.**c]** We summarize for all identified pollen-based food plants relevant background information in terms of vernacular name, pollen atlas(es) of reference, their earliest known use, their environmental preference and geographical region. **[I**.**d]** We summarize the environmental conditions (open vegetation vs. half open to closed forest) where the families of food plants are most commonly found. **[II**.**a]** We present a reference dataset that identifies the abundance of pollen-based human indicators from Latin America, based on a literature compilation by Flantua *et al*.^36^. **[II**.**b]** We guide the reader to the presence/absence of pollen-based human evidence from an extensive review of pollen-focused paleoecological references in Latin America. **[II**.**c]** We present datasets that include pollen-based human indicators based on single pollen types and human ‘indices’ that consist of combinations of pollen types that grouped together provide different degrees of evidence of human presence **[II**.**d]** We detail for all identified human indicators how the original studies assigned them to human presence, and we suggest an indication of the level of evidence (weak-neutral-strong) these indicators and indices provide when encountered in fossil pollen records. In conclusion, by providing comprehensive reference datasets, guiding the readers to pollen morphological illustrations and paleoecological literature reviews, summarizing background information on food plants, and offering datasets on human indicators, we offer valuable resources for a broad range of researchers interested in human history.

## Food plants of global relevance

### Historical shift to cultivated crops and loss of genetic diversity in human diets

Initially, food plants were gathered from the wild; however, with the advancement of agriculture, certain wild plants were domesticated. This domestication process, spanning thousands of years and involving over 150 generations of farmers worldwide, transformed the human diet. The transition from reliance on wild foods to predominantly cultivated crops began during the middle Holocene, notably in the Middle East before spreading to other regions, and has continued to evolve over time^37,38^^,page17^. No significant new plants or animals for human diets have been domesticated since this transition. In various continents, such as Australia, North America, and Amazonia, the displacement of native peoples from their land has resulted in the destruction of much of the accumulated knowledge and practices of over 60,000 years^37^^,page32^. During the late nineteenth century, Mendelian genetics were applied to breeding crop plants, leading to the development of new, higher-yielding varieties during the Green Revolution. However, this approach often resulted in the creation of monocultures of genetically identical plants, which reduced genetic diversity and increased reliance on a limited number of crops^37^^,page,67,68^. As a consequence, out of the 6000 plant species historically consumed by humans, only nine are now commonly consumed worldwide, with rice, wheat, and maize alone providing 50% of all calories^37^^,page8^. The level of genetic uniformity observed in these three major crops has never been experienced before in history^37^^,page2^.

### Rapid changes in human diets: historical shifts and overlooked crop varieties

The changing human diet has undergone more rapid changes in the last 150 years than ever before, with global trade, technology, and corporate power driving dietary shifts across the world^37^. For example, emmer wheat, which was once a widely cultivated grain in ancient Egypt, Mesopotamia, and Greece, is now on the brink of extinction^37^^,page63^. Similarly, the history of citrus trees is complex, with all commercial citrus fruits having three main ancestors: the mandarin, the pomelo, and the citron. Hybridization among these ancestors has led to the development of oranges, lemons, limes, and grapefruit, which are widely consumed today^37^. However, it is important to note that past crops of food plants, which may have been significant in human diets, could be overlooked in older pollen records, while crops bred in more recent centuries may not be present in older sediments. Therefore, isolated spikes in pollen records that potentially include food plants may provide hints of local crop systems, and careful attention should be paid to such events to better understand historical changes in human diets.

## Human indicators of Latin American relevance

Pollen-based paleoecology in Latin America has revealed a rich history of human-environment interactions^24,39^. Human indicators based on fossil pollen are instrumental in providing evidence of the magnitude and extent of human activities by the presence of pollen of food crops, anthropogenic weeds, and plants with differential uses in entnobotanical sense, among others. The complex interplay between humans and their environment has been reflected in studies reconstructing pre-Columbian land-use practices (e.g.^40,41^) and first appearances of introduced species (e.g.^24^), for instance.

To accurately gauge the combined impact of human activities and climate change on the environment in Latin America, an understanding of the extent of human activities is crucial. Currently, there is a lack of a comprehensive overview of human indicators identified from fossil pollen records. This shortfall can result in several issues, including:

1. Underestimation of human impact: If human indicators are overlooked or not well understood, the extent and nature of human activities, such as agriculture, deforestation, and resource extraction, may be underestimated.
2. Insufficient knowledge about human indicators can lead to inaccurate reconstructions of past environments and their ecological conditions, affecting our understanding of environmental and ecological change over time.
3. Misinterpretation of data: A lack of familiarity with human indicators can lead to misinterpretation of data from fossil pollen records, leading to incorrect conclusions about human-environment interactions.
4. Limited ability to make informed decisions: Without a comprehensive understanding of human indicators, it becomes challenging to make informed decisions about sustainable land use practices, conservation efforts, and the management of natural resources.

Human indicators can be based on single pollen types or a combination of pollen types, and have different degrees of evidence. Single pollen types, such as adventives and apophytes, can serve as direct evidence of human presence. Adventives, which are pollen grains from introduced or cultivated plant species, indicate human activities such as agriculture or horticulture. Cultivated crops and weeds can be distinguished based on their pollen types (although they are notoriously similar), providing information about land use practices and the selection and management of certain plant species by humans. Apophytes, on the other hand, represent plants that have adapted and proliferated in response to human disturbance, reflecting the ecological changes caused by human activities. However, the significance of single pollen types alone may be limited in capturing the complexity of human impact. Human indices, which are combinations of specific pollen types, can offer a more comprehensive approach by considering the co-occurrence and patterns of multiple pollen taxa associated with human activities. These indices allow researchers to identify distinct ecological signatures resulting from human presence, such as the introduction of new plant species, the alteration of vegetation composition, or the creation of open landscapes through deforestation or land clearance.

Based on the presence and abundance of specific pollen types or combinations thereof, researchers can gain a more nuanced understanding of the ecological consequences of human activities in the past. This approach enables not only the identification of different degrees of human evidence, ranging from weak to strong, but also allows for a more comprehensive reconstruction of past human activities and their ecological consequences. This knowledge contributes to our broader understanding of human history and provides valuable insights into the intricate relationships between humans and the environments they inhabit.

## Methods

### The identification of food plants of global relevance based on Van Wyk

**Fig. 1:**
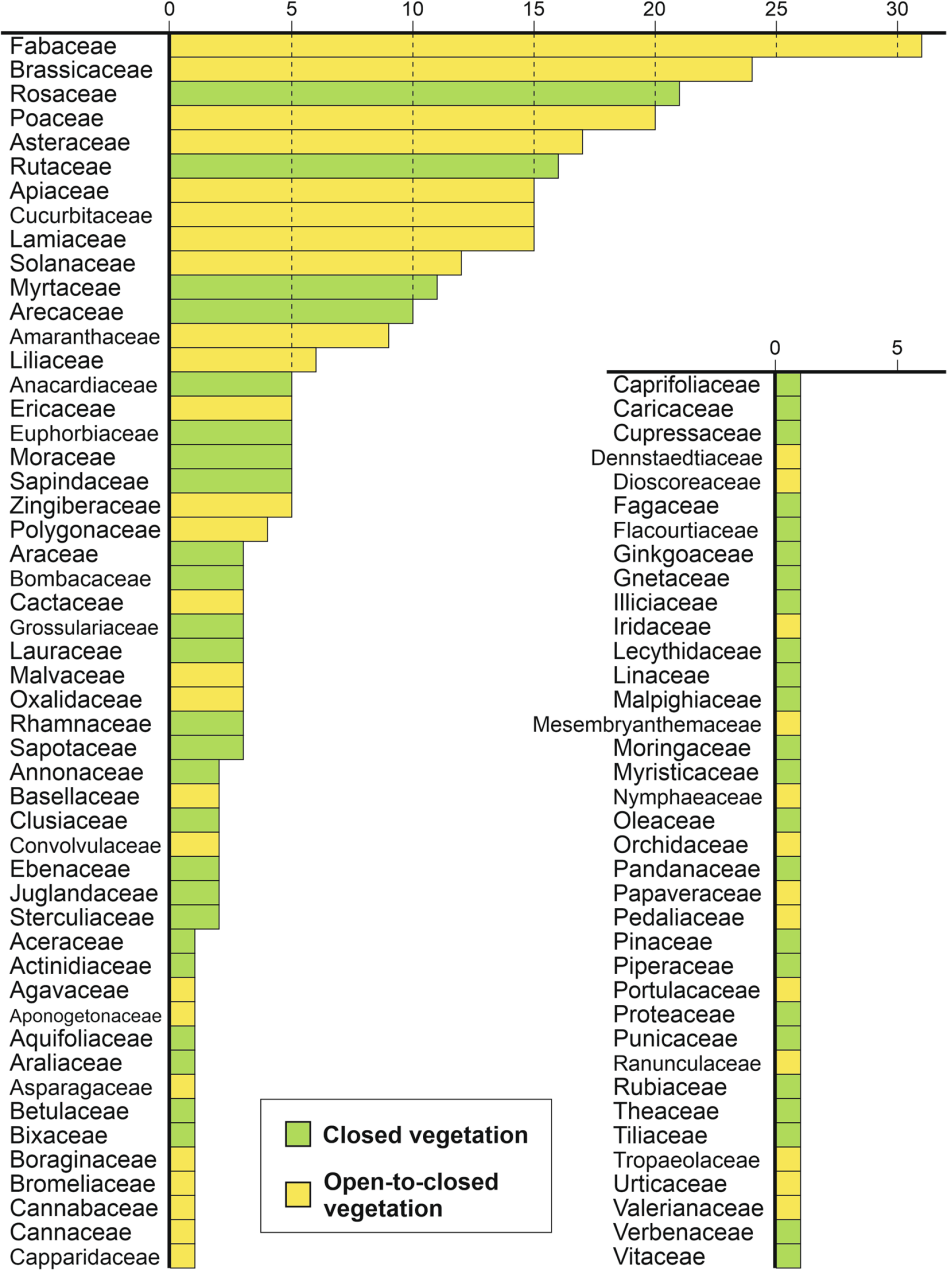
Frequency of food plants (n = 391) per family and their preferred vegetation setting.

A variety of literature exists on global compilations of food plants, such as^42–44^. Van Wyk^35^ represents one of the most extensive compilations of global food plants covering the various categories of plant use, including cereals, nuts and seeds, vegetables and spices. From this illustrated guide, we identified 391 food plants of global relevance, representing 88 different families and 273 different genera (Fig. 1). We compiled metadata for each food plant with paleoecological relevance related to pollen identification level, environmental settings, and human history.

The compilation by Van Wyk^35^ is a collection of food plants of global relevance, while for the vast number of food plants of regional and local importance, regional literature should be consulted. Such compilations exist for instance for Germany^45^, the Netherlands^46^, Europe and the Near East^47^, Bolivia^48^, Ecuador^49^, and Colombia^50^. Much information is also hidden in the vast archaeological and ethnobotanical literature. For example, the Spanish book ‘Atlas de polen de plantas utiles y cultivadas de la Amazonia Colombiana’ (Pollen atlas of useful and cultivated plants in the Colombian Amazon region^51^) shows many pollen morphological illustrations. The book ‘Comparative ethnobotanical studies of the Amerindian groups in coastal Ecuador’^49^ lists many plant taxa and their use as food plants. The numerous medical and ritual uses are also of high relevance but these categories of plants are beyond the scope of this global overview. Some cultivants that are now cultivated at large scales are not mentioned by Van Wyk.

### Pollen morphological illustrations of food plants

Van Wyk’s^35^ compilation illustrates 391 selected food plants. To facilitate the linkage between these food plants and pollen grains identified in fossil pollen records, we conducted a review of 10 pollen atlases and manuals that cover a wide taxonomic range and geographic regions (Table 1). Our review provides references to both widely used and less-known pollen atlases, some on which are still available as a hardcopy. Out of the 391 species of food plants, 280 species were illustrated in the selected pollen atlases and manuals. In total 111 species, including 66 genera, were missing. We anticipate that many of the missing genera can be found in the dispersed pollen morphological literature (see^52^; an update to this compilation in preparation). Our dataset serves as a contribution to the identification of pollen grains from food plants and recognizing evidence of human presence in fossil pollen assemblages. Such information is currently very scattered. We recommend a more comprehensive study using freshly collected pollen samples and a uniform preparation and documentation method to better advance pollen-based assessments of human history in interaction with vegetation and plant food sources.

**Table 1:** Overview of selected pollen atlases.

Abbreviation	Pollen atlas title (Year)^citation^	Comments
**AM**	**Amazon pollen manual and atlas** (**1999**)^55^	Fossil pollen grains from a sediment core are shown
**BCI**	**Pollen and Spores of Barro Colorado Island** (**1991**)^56^	Fresh material has been studied to obtain pollen images.
**CA**	**Atlas de polen de plantas utiles y cultivadas de la Amazonia colombiana** *(****Pollen atlas of useful and cultivated plants in the Colombian Amazon region****)* (**1996**)^51^	Fresh material has been studied to obtain pollen images.
**CHI**	**An Illustrated Handbook of Quaternary Pollen and Spores in China** (**2016**)^57^	Fresh material has been studied to obtain pollen images.
**DRA**	**Genera Palmarum - The Evolution and Classification of the Palms** (**2008**)^58^	Fresh material has been studied to obtain pollen images.
**EUR**	**Leitfaden der Pollenbestimmung für Mitteleuropa und angrenzende Gebiete** *(****Guide to pollen determination for Central Europe and adjacent areas****)* (**2004**)^59^	Fresh material has been studied to obtain pollen images.
**JAP**	**Pollen flora of Japan** (**2011**)^60^	Fresh material has been studied to obtain pollen images.
**MWC**	**Pollen analysis** (**1991**)^8^	Fresh material has been studied to obtain pollen images.
**TAI**	**Pollen Flora of Taiwan** (**1972**)^61^	Fresh material has been studied to obtain pollen images.
**TH**	**Les palmiers: palynologie et systématique** *(****The palms: palynology and systematics****)* (**1970**)^62^	Fresh material has been studied to obtain pollen images.

Van Wyk´s compilation illustrates 391 selected food plants. To facilitate the linkage between these food plants and pollen grains identified in fossil pollen records, we conducted a review of 10 pollen atlases and manuals that cover a wide taxonomic range and geographic regions. Abbreviations of pollen atlases correspond to abbreviations in our datasets available at Figshare^54^.

### The identification of human indicators and indices in Latin America fossil pollen records

We reviewed 750 publications to compile an overview list of pollen-based human indicators covering all countries and ecosystems of Latin America. For every country in Latin America, we reviewed in detail a representative number of references until no new indicators were found. Also, countries where we identified more studies^36,53^, we reviewed a higher number of references. References were discarded when covering a time period that was too old (e.g. early Quaternary) or when full access to the original reference was not possible. For each identified human indicator, we summarized the reason why this pollen type or human index is considered as human presence, we suggest the degree of evidence it provides, and we referred to one or more references suggesting the human indicator/index. We identified 212 and 95 human indicators based single-pollen types and human indices, respectively. For each human index, we specify the country/region where the human index was suggested for.

## Data Records

The full dataset “FOOD PLANTS AND HUMAN INDICATORS” is stored at Figshare^54^ and includes tabs (spreadsheets) with the lists of global food plants and atlases, the Latin American human indicators and indices, and the LAPD inventory of modern and fossil pollen records, and corresponding literature overviews. The individual tabs are described below.

**TAB 1 GLOBAL FOOD PLANTS: Food plants and corresponding metadata information relevant to palynological research**

The presented overview aims at a better identification of food plants on the basis of fossil pollen. The entire list consists of the information as provided by Van Wyk^35^ but compiled easily accessible for paleoecologists. The structure of the spreadsheet is alphabetically by family and then by genus, and includes information for each food plant, concerning natural source area, where the plant was introduced, and when available, its earliest use, and reference to illustrations in the pollen atlases. For each genus, we indicate at which taxonomic level the pollen grain can be identified which allows to develop an understanding how relevant the presence of an ‘undifferentiated’ family in a pollen record potentially may be as an indicator of a source of food.

Metadata:

**Latin genus and species** (**column A**)**:** Latin genus and species names.

**Family** (**Column B**)**:** Family name follow Van Wyk^35^.

**Vernacular name** (**Column C**)**:** Vernacular name(s) in English follow Van Wyk^35^.

**Geographic regions** (**Column D**)**:** Current regional source of food plant. The symbol ‘>’ means the food plant was introduced into a new area. EUR: Europe, Nam: North America, W&C-Asia: West & Central Asia.

**Van Wyk page number of illustration** (**Column E**)**:** The page number in Van Wyk^35^ with an illustration of the food plant.

**Earliest known use** (**Column F**)**:** Following Van Wyk^35^, the earliest known use is reported in ‘thousands of years before present’ (ka), e.g., 6ka. The abbreviation *‘ant*’ means ‘since antiquity’.

**Illustration in pollen atlases** (**Column G**)**:** YES: illustration at species level of pollen morphology found. NO: No illustration found. X: No illustration at the species nor genus level.

**Pollen atlases provide illustration of different species of the genus** (**Column H**)**:** An illustration of the genus of the food plant can exist but from a different species. YES: Illustration from different species. NO: No illustration.

**Pollen atlas used,** **plate/page number: illustration number** (**Column I**)**:** XX) caps refer to the pollen atlas used (Table 1), and is followed by plate number/page number, and illustration number, e.g., EUR 251:15–18; CHI 6:1–6. An asterisk (*) means it is an illustration of the species concerned, e.g., EUR 485:3*-5*. in all other cases an illustration should be used of a different species belonging to the genus concerned. Pollen atlases abbreviations: AM^55^, BCI^56^, CA^51^, CHI^57^, DRA^58^, EUR^59^, JAP^60^, MWC^8^, TAI^61^, TH^62^.

**Comments** (**Column J**)**:** Additional comments.

### Pollen-based human indicators and indices in fossil pollen records in Latin America

The presented overviews include information for each human indicator and index referring to the way they were described by the corresponding reference. The structure of the spreadsheets is alphabetically by human indicator (single-pollen types) or index (combination of human indicators). For each one, we summarize their human impact/presence description with categories, their corresponding references, the degree of evidence (neutral, weak, strong) we suggest they indicate based one or more single-counted pollen grains, and the likely level of taxonomic identification (i.e. species, genus, family).

**TAB 2 - LAPD HUMAN INDICATORS: List and descriptions of identified human indicators as found in the literature list of the Latin American Pollen Database**

Metadata:

**Pollen type** (**column A**)**:** Name of pollen type for human indicator as given by reference(s).

**Common name** (**Column B**)**:** Common name as given by reference(s).

**Disturbance** (**Column C**)**:** When TRUE, the pollen type is suggested to be an indicator of disturbance.

**Cultivation** (**Column D**)**:** When TRUE, the pollen type is suggested to be an indicator of cultivation.

**Edible parts** (**Column E**)**:** When TRUE, the pollen type is suggested to reflect plants with edible parts.

**Arboreal resources** (**Column F**)**:** When TRUE, the pollen type is suggested to be an arboreal resources, such as for timber and wood materials.

**Medical** (**Column G**)**:** When TRUE, the pollen type is suggested to have medical uses.

**Exotic species** (**Column H**)**:** When TRUE, the pollen type is suggested to be an exotic (introduced) type.

**Other** (**Column I**)**:** When TRUE, the pollen type is suggested to be in a different category of human indicator. See more in the column Description.

**Main category** (**Column J**)**:** Pollen types can be suggested to fall in different categories of human indicators but often one main category prevails.

**Evidence** (**Column K**)**:** Relying on our background knowledge of pollen morphology in Latin America, we facilitate an arbitrary indication of the level of evidence by the use of a single counted pollen grain of the corresponding pollen type.

**Description** (**Column L**)**:** Expanded details on the information given on the pollen type as taken from the reference(s).

**Example references** (**Column M**): One or more references that mention the pollen type as a human indicator.

**Additional information** (**Column N**)**:** Additional information provided by^63^.

**Family** (**Column O**)**:** Family name as assigned by^63^.

**Likely pollen identification level** (**Column P**)**:** Likely taxonomic level of identification (species, genus, family) based on the morphological features.

**TAB 3 - LAPD HUMAN INDICATORS FREQUENCY: List and frequency of human indicators as found in the literature list of the Latin American Pollen Database**.

List is ordered according to the number of times the indicator was found to be mentioned in the references that reported human indicators (n = 348). The references representing each human indicator can be found in Tab. 5. Note 1: Some human indicators can occur multiple times, but specifics differ (e.g. *Pinus* as introduced taxa or disturbance indicator). Note 2: We used the original taxonomic names as provided by the reference, i.e. taxonomic harmonisation^64^ has not been done.

Metadata:

**Human indicator** (**column A**)**:** Human indicator by pollen type, fire, or other type of indicator

**Specifics** (**column B**)**:** Additional comments

**Number of references** (**column C**)**:** Number of times the human indicator was found in a reference.

**%** (**column D**)**:** The percentage (%) is calculated based on the number of ‘positive’ references specifying human indicators.

**Fig. 2:**
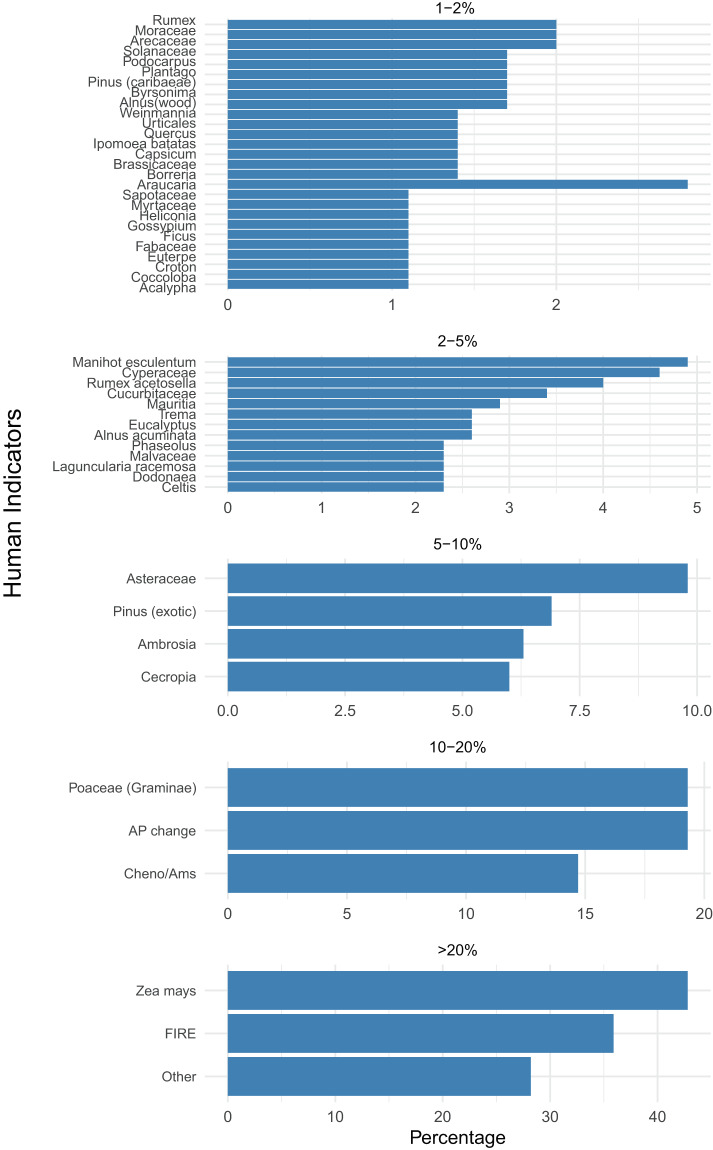
Distribution of human indicators in fossil pollen records in Latin America. Horizontal bar plots depict the distribution of human indicators in percentages identified in fossil pollen records. The human indicators are grouped into percentage ranges based on their frequency of occurrence in the literature. Each group is displayed in a separate facet, allowing for a detailed exploration of the distribution pattern across different percentage ranges. Group representing human indicators occurring < 1% is not shown but see our Code Availability.

**Fig. 3:**
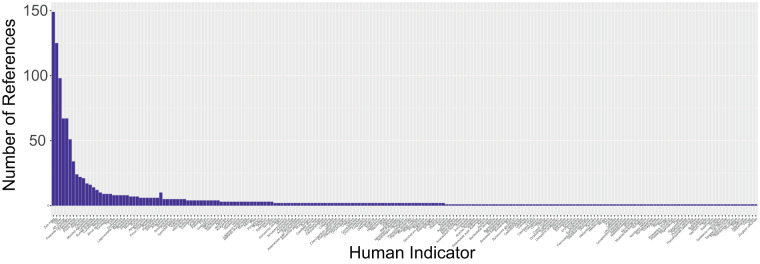
Number of times each human indicator was identified in references in Latin America on (fossil) pollen records. The exact values are found in Tab. 3. The figure is meant as illustrative purpose to indicate the long tale of rare human indicators.

Each human indicator is present at least once across the 750 references checked. Figure 2 shows the percentage of ‘positive’ references (at least one human indicator present, n = 348) mentioning that pollen type as human indicator. Numerous human indicators are found in <1% of the reviewed references with human indicators (n = 160, not shown in Fig. 2) creating a long tail of rare human indicators (Fig. 3).

**TAB 4 - LAPD HUMAN INDICES: Identified human indices in references from the Latin American Pollen database**

Metadata:

**Original naming** (**column A**)**:** Naming or description given to the human index by the original reference.

**Original scope region** (**Column B**)**:** Region to which the human index is applied following the area of study of the reference.

**Pollen types included** (**Column C**)**:** Pollen types as included in the human index. Note that in some cases, the human index includes other human indicator types such as charcoal and fern spores.

**References** (**Column D**)**:** One or more references that mention the combination of pollen types as a human index. Note that this listing is not exhaustive, i.e. the same human index could have been used for other regions across Latin America.

**Fig. 4:**
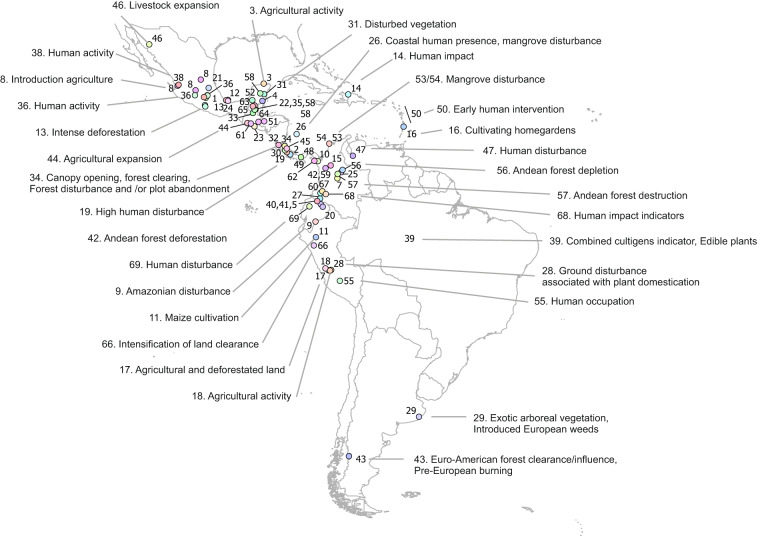
Locations of a selected number of references with human indices. Note: #39 covers several records in different regions across the Amazon; #50 represents several records across different islands in the Lesser Antilles; #53–54 represent several records along the Colombian coast. Map was created in ESRI ArcGIS^68^. Country polygons are provided by^69^. Full list and description are found in Tab. 4.

**Figure 4 (Column E**): Number refers to the map shown in Figure 4.

**Evidence** (**Column F**)**:** We facilitate an arbitrary indication of the level of evidence using the human index with the corresponding human indicators being counted once or more.

**TAB 5 - LAPD LITERATURE REVIEWED: Reviewed literature list from Latin America with identified human indicators per reference**

The overview lists the reviewed references obtained from the full literature list (Tab 6 and 7) of the Latin American Pollen Database (LAPD^36,53^) to identify for each reference if human indicators were described. Each row represents a reviewed reference and the identified human indicator as derived from the corresponding reference(s). Total number of human indicators including pollen types, charcoal, other indicators: 211. There are some slight differences from Tab. 2 (n = 212, only representing pollen types) as some pollen types we counted as 1 group, e.g. Asteraceae and *Laguncularia racemose*.

Metadata:

**Reference** (**column A**): Reference information as mentioned in Human indicators overview file (Column M) and Human indices overview file (Column D).

**HUMAN** (**column B**)**:** TRUE/FALSE if a human indicator is identified (column F-HE).

**FIRE** (**column C**)**:** TRUE/FALSE if there was mentioning of evidence of fire (charcoal) to be suggestive of humans.

**Other** (**column D**)**:** TRUE/FALSE if besides fire or pollen, other human indicators were mentioned (e.g. spores, changes in sedimentation rates, etc).

**AP change** (**column E**)**:** TRUE/FALSE if arboreal pollen % was suggested to have changed due to humans (i.e. deforestation, selective logging, compositional change).

**Human indicators** (**columns F-HE**)**:** Name of the human indicator of pollen types as mentioned by reference.

### Inventory of modern and fossil pollen records in Latin America and corresponding literature list

The LAPD literature list by Flantua *et al*.^36^ was compiled in an effort to create an extensive inventory of modern and fossil pollen records across Latin America, identifying over 4800 and c. 1400 datasets, respectively. We updated the full list of references and is now also made available at http://www.latinamericapollendb.com/^53^. Over 1700 references were identified from peer-reviewed and non-peer reviewed publications and chapters/books, Ph.D./Master theses, congress abstracts, reports, among others.

**TAB 6 - LAPD FULL INVENTORY: Inventory of modern and fossil pollen records in Latin America**

The inventory presents for each identified modern or fossil pollen record in Latin America their metadata information concerning location, chronology, depositional environment, proxies, and references, among others.

Metadata:

**LAPD_ID** (**Column A**)**:** Id number that refers to the overview file of references.

**LAPD89** (**Column B**)**:** Numerical count of datasets.

**VERSION** (**Column C**)**:** The list was updated during different periods since its creation. These versions refer to these periods with different funding.

**Record** (**Column D**)**:** Name of modern/fossil record

**Country** (**Column E**)**:** Country where record is/was located.

**Latitude** (**Column F**)**:** Decimal degrees

**Longitude** (**Column G**)**:** Decimal degrees

**Note_loc** (**Column H**)**:** Note on location and elevation, e.g. if estimated by use of Google Earth.

**Altitude** (**Column I**)**:** Elevation in meters above sea level.

**Modern_Paleo** (**Column J**)**:** If dataset is modern or fossil (paleo) pollen record

**Depth_m** (**Column K**)**:** If dataset is fossil pollen record, what is the depth of the record.

**Dataset type** (**Column L**)**:** Pollen inventory = fossil pollen record. Pollen surface sample inventory = modern pollen sample.

**Proxies** (**Column M**)**:** Which proxies are used with this record?

**Multiproxy** (**Column N**)**:** Is the record multiproxy: TRUE = 1; FALSE = 0

**Charcoal** (**Column O**)**:** Has charcoal been assessed with this record: TRUE = 1; FALSE = 0

**Environment** (**Column P**)**:** Information on the depositional environment.

**Ecosystem 1** (**Column Q**)**:** Information on the main ecosystem where record is located.

**Ecosystem 2** (**Column R**)**:** Information on the second most important ecosystem where record is located.

**Biome 1** (**Column S**)**:** Information on the main biome where record is located. Naming follows code naming as in^39^.

**Biome 2** (**Column S**)**:** Information on the main biome where record is located. Naming follows code naming as in^39^.

**No_control_points** (**Column U**)**:** Number of chronology control points

**Type of chronology control points** (Columns V-AH): Different types of chronology control points could have been used in fossil pollen records. TRUE = 1; FALSE = 0

**REF_C14** (**Column AI**)**:** Age reference of youngest radiocarbon dating (if present), i.e. BC, BP, AD.

**C14_MIN** (**Column AJ**)**:** Youngest age of dating of chronology control point (uncalibrated)

**MIN_error** (**Column AK**)**:** Error estimate of youngest age of dating of chronology control point

**C14_MIN** (**Column AL**)**:** Oldest age of dating of chronology control point (uncalibrated)

**MIN_error** (**Column AM**)**:** Error estimate of oldest age of dating of chronology control point

**REF_CalAge** (**Column AN**)**:** Age reference of youngest calibrated dating (if present), i.e. BC, BP, AD.

**time_min_BP** (**Column AO**)**:** Youngest recalibrated age of the record (recalibrated age), yr BP

**time_max_BP** (**Column AP**)**:** Oldest recalibrated age of the record (recalibrated age), yr BP

**Time_estim** (**Column AQ**)**:** Estimated age ranges estimated by S. Flantua based on publications. TRUE = 1; FALSE = 0.

**Additional notes** (**Column AR**)**:** Additional notes on age ranges.

**No_surface_samples** (**Column AS**)**:** Number of surface samples (n case of modern pollen sampling).

**Publ_dec** (**Column AT**)**:** Decade in which main reference was published.

**Publication year** (**Column AU**)**:** Year in which main reference was published.

**Publications** (**Column AV**)**:** Main references related to the pollen records

**Publication_ID** (**Column AW**)**:** ID of publication refers to full literature list on modern and fossil pollen records across Latin America (column B).

**Publication_ID** (**Column AX**)**:** ID of publication from which a PDF has been obtained.

**TAB 7 – LAPD FULL REFERENCE LIST: Full literature list on modern and fossil pollen records from Latin America**

The presented overview presents the list with full references with identified pollen records in Latin America (Tab. 6).

Metadata:

**REFERENCE** (**Column A**)**:** Full reference

**ID** (**Column B**)**:** ID of reference

**LAPD-SITE#:** When the reference has been used for a record in the modern and fossil pollen inventory (Tab. 6), then the number is mentioned here. (Note that there are references that have not been assigned yet to a modern or fossil pollen record).

## Technical Validation

We used a representative set of literature for the pollen grain illustrations aimed to represent a global coverage. This literature is the baseline for numerous publications and studies validating these sources of information throughout the years. We performed a thoroughly literature review to compile the human indicators of Latin America using 750 references obtained from the updated full literature list of the Latin American Pollen Database^36,53^. These references were compiled from all countries and ecosystems across the continent, in different languages and journals/reports/theses/etc.

## Usage Notes

Our comprehensive synthesis of food plants and references to the pollen morphology, and human indicators in fossil pollen records from a global and Latin American perspective, respectively, will aid in filling an important gap in global and continental-scale reconstructions of human presence and interaction with ecosystems. Our datasets of anthropogenic pollen indicators can be linked to the ‘raw’ fossil pollen data as obtained from Neotoma^14^ and PANGAEA^15^, after standardisation and taxonomical harmonisation. We recommend Flantua *et al*.^65^ for a workflow to standardise fossil pollen datasets and Birks *et al*.^64^ for approaches and tables at continental level to harmonise fossil pollen datasets. A resulting synthesis of the identification of anthropogenic pollen indicators would be essential for understanding human history, as it could further illuminate the development of agriculture, provide insights into subsistence strategies and diets of past societies, reveal relationships between human populations and their environments, and enables the tracing of the geographical distribution of food plants over time. It is important to note that the use of fossil pollen of food plants, and pollen grains reflecting human indicators, varies across regions. Therefore, our datasets specify geographical locations where possible (see discussion in^21^). By providing region and site-specific assessments of human indicators, researchers working with fossil pollen records can trace the food sources and vegetation changes associated with human activities in a more nuanced and localized manner.

Our comprehensive analysis of fossil pollen records from Latin America reveals a fascinating pattern in the distribution of human indicators. Among the 160 identified human indicators, only a few are frequently mentioned in the literature, such as *Zea mays*, changes in arboreal pollen and grass (Poaceae) percentages (Fig. 2). In contrast, a substantial portion of human indicators (20 indicators) is mentioned in only 2–10% of the reviewed papers with human indicators, and a similar number (25 indicators) is found in a mere 1% of the literature. The remaining human indicators are elusive for the diversity of human indicators found in Latin America, being mentioned in less than 1% of the papers (Fig. 3). This intriguing distribution of human indicators underscores the existence of a long tail of “rare” human indicators in fossil pollen records across the continent. While some human indicators have received considerable attention and recognition in paleoecological research (e.g. *Zea mays* and *Cecropia*), there exists a vast and relatively unexplored landscape of less frequently identified indicators. This finding suggests that human interaction with the environment is likely more diverse and nuanced than previously acknowledged. Moreover, these less frequently cited indicators could serve as critical pieces of evidence in deciphering specific regional or local patterns of human impact and cultural practices. Exploring more these rare human indicators in future research may shed light on lesser-known aspects of ancient human activities, subsistence strategies, and land use practices.

The identification of numerous rare human indicators has significant implications for future research and paleoecological studies. It highlights the need for a more comprehensive and interdisciplinary approach to fully understand the complexities of human-environment interactions in the past. In numerous occasions, we detected human indicators based on pollen types to have been combined with other evidence such as increased fire occurrence, changes in sedimentation rates/composition, and dung fungi, indicating the high degree of interdisciplinary approaches taken by researchers across the continent (Fig. 2). We do emphasize that not all human indicators are easily taxonomically identifiable (see our suggestion in Column P of the pollen indicators dataset) and some may be merged during taxonomical harmonisation (see^64,65^). An assessment of genera/families with high number of human indicators could nevertheless provide valuable insights.

Further integration of our continental synthesis with large-scale databases on fire and archaeological evidence (see^66,67^) would be essential for unambiguous evidence of human history. Continental-scale reconstructions of human-vegetation interaction are absent in Latin America but such synthesis would open up avenues research into topics such as temporal occurrence of human indicators over time (e.g. detect the earliest evidence of human presence or certain crops), shifting degrees of human impacts on vegetation, dietary patterns of ancient societies, deforestation dynamics, cultural exchange, and the interaction between climate change and human activities. By combining diverse disciplines and utilizing our synthesis of human indicators, future research can shed new light on the complex relationship between humans and the environment in Latin America’s past.

## Data Availability

Code to create Figs. 2–4 can be found here: https://github.com/HOPE-UIB-BIO/Global-Food-plants-and-LAPD-HI. Note: Small adjustments for aesthetics were made in an external illustration software.
